# Core regulatory circuitries in defining cancer cell identity across the malignant spectrum

**DOI:** 10.1098/rsob.200121

**Published:** 2020-07-08

**Authors:** Leila Jahangiri, Loukia Tsaprouni, Ricky M. Trigg, John A. Williams, Georgios V. Gkoutos, Suzanne D. Turner, Joao Pereira

**Affiliations:** 1 Department of Life Sciences, Birmingham City University, Birmingham, UK; 2 Division of Cellular and Molecular Pathology, Addenbrooke's Hospital, University of Cambridge, Cambridge, UK; 3 Department of Functional Genomics, GlaxoSmithKline, Stevenage, UK; 4 Institute of Translational Medicine, University Hospitals Birmingham NHS Foundation Trust, Birmingham, UK; 5 Institute of Cancer and Genomic Sciences, College of Medical and Dental Sciences, University of Birmingham, Birmingham, UK; 6 Mammalian Genetics Unit, Medical Research Council Harwell Institute, Oxfordshire, UK; 7 MRC Health Data Research, UK; 8 NIHR Experimental Cancer Medicine Centre, Birmingham, UK; 9 NIHR Surgical Reconstruction and Microbiology Research Centre, Birmingham, UK; 10 NIHR Biomedical Research Centre, Birmingham, UK; 11 Department of Neurology, Massachusetts General Hospital, Harvard Medical School, Charlestown, USA

**Keywords:** core regulatory circuitry, liquid and solid cancers, super-enhancers, cell identity

## Abstract

Gene expression programmes driving cell identity are established by tightly regulated transcription factors that auto- and cross-regulate in a feed-forward manner, forming core regulatory circuitries (CRCs). CRC transcription factors create and engage super-enhancers by recruiting acetylation writers depositing permissive H3K27ac chromatin marks. These super-enhancers are largely associated with BET proteins, including BRD4, that influence higher-order chromatin structure. The orchestration of these events triggers accessibility of RNA polymerase machinery and the imposition of lineage-specific gene expression. In cancers, CRCs drive cell identity by superimposing developmental programmes on a background of genetic alterations. Further, the establishment and maintenance of oncogenic states are reliant on CRCs that drive factors involved in tumour development. Hence, the molecular dissection of CRC components driving cell identity and cancer state can contribute to elucidating mechanisms of diversion from pre-determined developmental programmes and highlight cancer dependencies. These insights can provide valuable opportunities for identifying and re-purposing drug targets. In this article, we review the current understanding of CRCs across solid and liquid malignancies and avenues of investigation for drug development efforts. We also review techniques used to understand CRCs and elaborate the indication of discussed CRC transcription factors in the wider context of cancer CRC models.

## 1. Introduction

Programmes involved in the control of gene expression governing cell state, cell state transitions and cellular identity across cell types or lineages have not been comprehensively defined. However, multiple efforts encompassing a myriad of differentiation models have shed light on the mechanisms regulating these developmental programmes [1–5]. These programmes are controlled by a small set of tightly regulated transcription factors (TFs) and/or *de novo* fusion chimeric TFs, forming core regulatory circuitries (CRCs). These CRCs control lineage-specific flow of information for gene expression [6–8]. Mechanistically, these core regulatory TFs (CR TFs) can control the placement of acetylation deposits around an array of CR TF binding motifs by recruiting acetylation writers, readers and erasers, thereby creating super-enhancers (SEs) [9]. SEs are broad, spatially co-localized enhancer regions that recruit dense transcriptional machinery. SEs are disproportionately larger than most enhancer domains and contain close to 40% of enhancer-associated factors (including epigenetic machinery), while comprising only 3–5% of enhancer regions [10]. CR TFs drive cell identity by binding to SEs associated with lineage identity imposing genes, often oncogenes [6,8,10–12]. CR TFs self-regulate and, they inwardly bind to their own regulatory regions and mutually regulate within the CRC, forming a cross-regulated feed-forward loop [6]. Research efforts to date have focused on understanding components of CRCs and their roles in multiple cell types, including embryonic stem cells (ESCs), induced pluripotent stem cells (iPSCs) and multiple cancer cell types [13–15]. In ESCs, CRC TFs including OCT4, SOX2 and NANOG regulate themselves and each other [10,14]. These CRC TFs dominate the transcriptional programmes governing stem cell self-renewal, pluripotency and cell fate [10,14]. Expression of this network of CRC TFs, with the addition of the proto-oncogene C-MYC, was sufficient to reprogramme somatic cells into iPSCs [16]. Similar efforts in cancers have brought into focus tumour dependencies and regulatory diversity and, in some cases, addiction to regulatory circuitries [15]. Further, SEs, as components of CRCs, are linked to regions of somatic genetic alterations such as focal amplifications in cancers and disease linked-SNPs [17,18]. SEs can also reinforce the expression of factors indicated in tumour development and progression [11].

An important step in understanding the role of CRCs in cancers is the systematic reconstruction of CRCs both in development and cancer. The reconstruction of CRCs for a cell type requires SE maps (usually indicated by high levels of a H3K27ac histone signature), core TF binding data, their putative binding sites in the SE regions and their extended, genome-wide, regulatory network [6,19]. To that end, Saint-André and colleagues reconstructed and predicted CRC models using a CRC mapper programme for 75 human cell and tissue types [6]. Huang and colleagues developed a dbCoRC database which, in addition to archiving CRC information, interactively reconstructs CRCs for over 230 human and mouse cell lines or primary tissue, inclusive of 79 cancer cells and tissues [19]. This database provides cell-type specific information about SEs, CRC models, putative binding sites for TFs identified in target gene SEs, and TF expression patterns [19]. Other resources such as dbSUPER also provide a comprehensive map of SEs identified in more than 100 cell types, which may be used to complement CRC model data [20]. The next step beyond CRC reconstruction in cancers is understanding the cellular and molecular mechanisms of divergence of constitutive developmental programmes in a background of genetic aberrations [6]. The inference of the underlying transcriptional networks that regulate physiological and pathological states is likely to inform these mechanisms of diversion and enhance our understanding of both physiology and disease. Put together, it is reasonable to propose that understanding the role of CRCs in cancers will facilitate the dissection of identity-conferring programmes and lead to a better understanding of their deregulation in cancers, potentially informing drug development and re-purposing strategies [15,21,22]. In this article, we review the present knowledge of CRCs across a multitude of solid and liquid cancers, and the current evidence for leveraging this information for therapeutic gain. We then attempt to elaborate the indication of discussed CRC TFs, in a wider range of cancer cells and tissues using the dbCoRC database. Finally, we describe current methodologies used to understand CRCs.

## 2. CRCs in a multitude of solid and liquid cancer types

**Figure 1.**
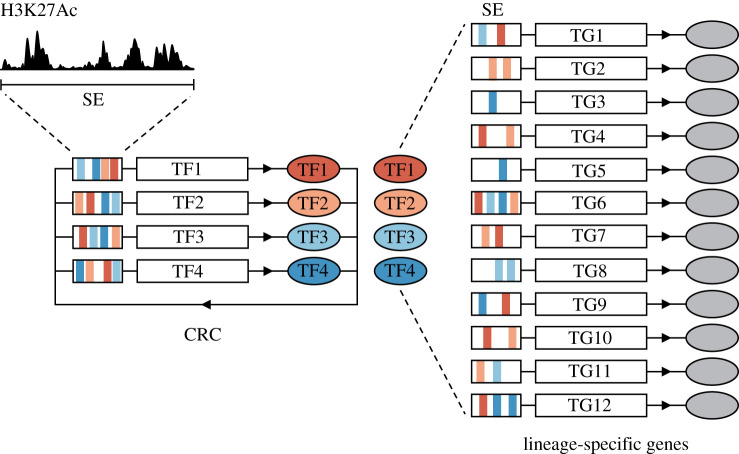
Core regulatory circuitry (CRC) constitutes a network that can confer lineage-specific gene expression. Core regulatory transcription factors (CR TFs) self-regulate and regulate the expression of other CR TFs in a cross-regulated feed-forward loop. Super-enhancers (SEs) contain CR TF binding sites and marked H3K27ac deposits. CR TFs, in turn, bind to the regulatory regions of a network of target genes (TGs) including lineage-specific genes that drive cell identity. TF, transcription factor; TG, target gene; SE, super enhancer. TF binding motifs (for TF1, TF2, TF3 and TF4) are depicted as rectangles in red, pink, light blue and dark blue, respectively.

In this section, we address the role of CRCs in controlling the flow of information that governs identity-conferring programmes in a multitude of solid and liquid cancer types (figure 1).

### 2.1. Neuroblastoma

Neuroblastoma (NB) is a solid malignancy derived from multipotent neural crest cells (NCCs) and contributes to 15% of cancer-related mortality in children [23]. Recent studies have defined the presence of two interconvertible types of NBs regulated by CRCs; committed adrenergic (ADRN) and neural crest migratory (or mesenchymal; MES) [12,24]. Though both cell populations are oncogenic [24], the latter type displays greater therapeutic resistance and encompasses the majority of relapsed tumours [25].

The Notch signalling pathway is the driver of motile MES identity, consistent with a mesenchymal phenotype. MES CRCs include the NOTCH receptors and cofactors, *NOTCH2* and *MAML2*, respectively, which are associated with SEs and drive an array of NOTCH target genes including *HES1* [24,26,27]. Members of the CRC-regulating MES state, namely, the NOTCH family, NOTCH1, NOTCH2 and NOTCH3, can initiate transdifferentiation to the ADRN state through H3K27ac landscape remodelling [24] and hence control maintenance of the MES state. However, the intracellular domain of NOTCH3 is the strongest inducer of reprogramming towards the MES state. Induction of the NOTCH3 intracellular domain leads to *de novo* establishment of SEs at *NOTCH2* and *MAML2* loci as well as the deposition of H3K27ac at the promoter regions of *JAG1, NOTCH1, NOTCH3* and *HES1* [24].

The CRC regulating the ADRN subtype in NB comprises PHOX2B, HAND2, TBX2, ISL1, ASCL1 and GATA3, whose effects are amplified by MYCN and LMO1 [25,28–30]. The most recent addition to this circuitry, ASCL1, a bHLH transcription factor implicated in NB cell growth and differentiation arrest, is directly regulated by LMO1, MYCN and other members of the CRC [31]. Similarly, ASCL1 directly regulates the expression of other genes in this CRC, forming an auto-regulatory loop [31]. Other members of this CRC, including GATA3, a biomarker linked to the proliferation of NB cells and self-renewal capacity [32], is downregulated following retinoic acid (RA) treatment, inhibiting tumourigenicity [32,33]. In addition, ISL1 positively regulates cell cycle genes and represses genes associated with differentiation (e.g. RA receptors, *CDKN1A* and *EPAS1*) [34].

The events leading to the oncogenic capacity and specificity of both ADRN and MES NB subtypes during development are still unknown. However, recent work by Soldatov and colleagues, which profiled gene expression during mouse neural crest development, may provide insights into the timing of NB oncogenesis. Single-cell RNA sequencing identified a novel bipotent cell type, a dual fate progenitor expressing both Phox2b and Prrx1, late in the differentiation cascade of NCCs [35]. As discussed, PHOX2B is expressed in ADRN subtypes while PRRX1 is MES-specific, and its overexpression is sufficient to convert ADRN to MES subtypes [24,25]. The existence of these dual progenitors could indicate they are upstream of the oncogenic event leading to the formation of both MES and ADRN NBs, and that further characterization of the complex SEs regulating cell fate decisions at this stage will be likely to inform NB biology. Table 1 summarizes examples of CRC TFs discussed in this section.

**Table 1.** Summary data of relevant CRC TFs identified in the indicated malignancies. In this table, cancer type and examples of subtype, subgroups, cliques or modules identified have been summarized. Further examples of CRC TF identified in each subtype, group, module or clique have been provided.

cancer	subtypes, subgroups or identified modules	examples of identified CRC TFs
neuroblastoma [24,31]	MES	NOTCH receptors and cofactors including NOTCH2 and MAML2
	ADRN	PHOX2B, HAND2, TBX2, ISL1, ASCL1 and GATA3, MYCN and LMO1
glioblastoma [36]		KLF4, ERG1, Notch pathway and SOX2
rhabdomyosarcoma [9]	Pan-RMS	MYOD1 and MYOG
** **	FP-RMS	PAX3-FOXO1, MYCN, SOX8, MYOD1 and MYOG
** **	fusion–negative RMS	PAX7 and AP1 family
** **	normal muscle-specific (NMS)	Nur77 and MEF2D
renal cell carcinoma [37]		PAX8
liposarcoma [38]	myxoid (MLPS)	FUS-DDIT3
** **	de-differentiated (DDLPS)	FOSL2, MYC and RUNX1
prostate cancer [39]		AR and ERG
gastrointestinal stromal tumour (GIST) [40]		FOXF1 and ETV1
medullablastoma [21]	group 3	HLX and LHX2
** **	group 4	LMX1A and LHX2
chronic lymphocytic leukaemia (CLL) [41]	CLL-2 (clique 2)	PAX5, ETV6, TCF3, IRF2, MEF2D, ELF1, KLF13, JUND, FOXP1, IRF1 and IRF8
** **	CLL-11 (clique 11)	PAX5, ETV6, TCF12, IRF2, RARA, NFATC1, KLF12, JUN, RUNX3 and FLI1
T-cell acute lymphoblastic leukaemia (T-ALL) [42]		TAL1, GATA3 and RUNX1

### 2.2. Glioblastoma

Glioblastoma (GBM) is the most common primary malignant brain tumour in adults and harbours distinct heterogeneous populations of tumour cells [43]. Earlier studies identified CRCs comprising the POU3F2, SOX2 and SALL2, OLIG2 TFs whose activities reprogrammed differentiated GBM cells into induced tumour propagating cells (TPCs). These TPCs have stem-like properties, are capable of tumourigenesis and display unique SE landscapes [43–45]. A target gene of this network is RCOR2, which forms a protein complex with LSD1, a histone methyltransferase. The RCOR2/LSD1 complex replaces OLIG2 in the reprogramming cocktail towards TPC [44]. Notably, most of these genes are involved in the maintenance of neural stem cell (NSC) identity during development. Expression of Pou3f2 (Brn2) was shown to be sufficient to convert astrocytes into neural progenitors in mice, similar to its role in the formation of TPCs [46]; SOX2 and OLIG2 are involved in maintaining the identity and replication potential of neural progenitors [47,48].

In a study conducted on glioblastoma stem cells (GSCs), NOTCH1, SOX2, SALL2, POU3F and OLIG2 blocked differentiation in GSCs, confirming the observations made in GBM by Suvà and colleagues [44,45]. Although the similarities and differences between induced TPCs and GSCs is not clear, it may be possible to propose that cells with self-renewal and tumourigenesis capacity can be identified in GBM or induced from differentiated GBM. Building on these observations, in a more recent study, Riddick and colleagues compare the global gene expression pattern of GSCs and NSCs during *in vitro* differentiation [36]. This group revealed a substantial overlap between the regulatory landscape of GSCs and NSCs. Further, in addition to the identification of important transcriptional regulators of GSC and NSC biology, such as SOX2, OLIG2, DLL, NOTCH and HES1, there were other significant observations. First, GSCs akin to NSCs express SOX2, Nestin and CD133, and demonstrate self-renewal and multi-potency while sharing common yet deregulated developmental pathways with NSCs including AKT, RAS, NOTCH, BMI-1 and WNT [36,49–53]. Second, the binding signature of TFs to differentially expressed genes was used to reconstruct a CRC centred on KLF4, a TF involved in activation of DDL1, NOTCH1 and SOX2 [36]. The overexpression of KLF4 in both GSCs and NSCs blocks differentiation and reduces proliferation [36,54]. In GSCs, KLF4 is regulated by ERG1 and sits downstream of STAT3 in the PI3K pathway [36].

Finally, consistent with potential plasticity of cell identity, glioblastomas can be reprogrammed towards mesenchymal lineages by the synergistic activity of initiators and master regulators, including STAT3 (downstream of PI3K activity) and CEBPB. Ectopic expression of these genes in NSCs reprogrammes these cells towards the mesenchymal lineages, and their expression in tumours is predictive of poor clinical outcomes, consistent with promoting motile phenotypes in these cells [55]. Table 1 summarizes examples of CRC TFs discussed in this section.

### 2.3. Rhabdomyosarcoma

Childhood rhabdomyosarcoma (RMS) is the most common soft tissue sarcoma in paediatric patients [56]. RMS oncogenesis relies on the expression of myogenic TFs [57], generating at least four identified CRCs in RMS tissue and cell lines: (i) a pan-RMS CRC defined by expression of MYOD1 and MYOG; (ii) a fusion-positive RMS (FP-RMS), which includes FOXO1 (SEs regulating *PAX3-FOXO1* or *PAX7-FOXO1*) and MYCN; (iii) a fusion-negative RMS including PAX7 and the AP1 family of TFs; and (iv) a normal muscle-specific CRC with TFs expressing Nur77 and MEF2D [58,59].

The FP-RMS module is formed by a t(2:13)(q35:q14) translocation forming a *PAX3-FOXO1* fusion gene, which functions as a primary oncogenic driver [9]. A consistently high-scoring H3K27ac signal and open chromatin structure was identified in the SE regions of *SOX8* in primary FP-RMS samples. More detailed investigation revealed that PAX3-FOXO1 positively regulates MYOD1, MOYG and SOX8 in a feed-forward mechanism [9].

MYOD1 and MYOG lead a pro-myogenic programme in RMS, while SOX8, a regulator of early neural crest development, displays anti-myogenic functions and opposes the ability of these factors to complete muscle differentiation [60]. Crucially, it is through the binding of PAX3-FOXO1 to SEs of *SOX8* and subsequent activation of SOX8 expression that this fusion protein can exert its anti-differentiation activity on these cells [9]. In conclusion, MYOD1 and MYOG are drivers of the myogenic programme, which is opposed by PAX3-FOXO1 via binding to the SE of *SOX8*.

The transcriptional interaction between SOX8, MYOD1 and MYOG is also interesting. Disruption of either MYOD1 or MYOG results in dramatic transcriptional downregulation of *MYOD1, MYOG, SOX8* and other TFs. Conversely, SOX8 is highly overexpressed in FP-RMS tumours, and SOX8 disruption leads to upregulation of *MYOD1* and *MYOG* in FP-RMS, suggesting a negative regulatory mechanism [9]. In conclusion, the FP-RMS CRC model includes feed forward (PAX3-FOXO1 and MYOD1, MYOG and other TFs) and negative feedback (SOX8) mechanisms [9,61].

In a more recent publication, Gryder and colleagues further dissect the CRC of FP-RMS and put forth a detailed mechanistic view of the chromosomal translocation that leads to hijacking of the *PAX3* promoter by *FOXO1* SE [62]. This group demonstrates that the SE of *FOXO1* interacts with smaller intergenic and intronic enhancers of *FOXO1* and *PAX3* promoter*.* In the stepwise developmental programme of skeletal muscle*,* PAX3 activates MYOD1 through *MYOD1* SE, but MYOD1 does not upregulate *PAX3*, and wild-type PAX3 enhancers are silent while MYOD1 and MYOG promote differentiation in late myogenesis [62]. By contrast, upon *FOXO1* SE translocation to regulate *PAX3* in FP-RMS, MYOD1, MYOG and MYCN can also bind to and drive this SE*.* This leads to the continuous expression of *PAX3-FOXO1* in late stages of myogenesis and halting of FP-RMS tumours in an undifferentiated state. These newly formed ‘miswired’ enhancer elements fuel the pathological diversion from normal skeletal muscle development in FP-RMS [62]. Table 1 summarizes examples of CRC TFs discussed in this section.

### 2.4. Renal cell carcinoma

Renal cell carcinoma (RCC) is a heterogeneous cancer accounting for 2% of all cancer cases [63]. Clear cell RCC (ccRCC) is the most common subtype of this disease (greater than 80% of all cases) and the main cause of RCC mortality. ccRCC harbours truncal mutations in the *VHL gene* (von Hippel-Lidau tumour suppressor) implicated in activation of TFs such as HIF1*α* and HIF2*α* that are involved in angiogenesis, metabolism and cell death [64]. However, consistent with the Knudsen's two-hit genetic alteration hypothesis, the addition of a second genetic alteration in mTOR pathways or chromatin modifiers is also required for induction of ccRCC [65]. In a recent study, PAX8, a cell-autonomous transcriptional activator, was identified as a potential CRC oncogenic driver in RCC, which may be independent of *VHL* alteration status [37]. *PAX8* knockdown in an array of RCC cell lines revealed a network of over 460 genes including those involved in metabolism, kidney cell fate, proliferation and the process of tumourigenesis (e.g. kidney-specific cadherins, claudins and cell cycle genes) under PAX8 regulation. One key difference between PAX8 regulation of metabolic genes compared with its other targets was the prevalence of H3K27ac. Specifically, cell cycle and metabolic pathway genes gained H3K27ac marks indicating that they were enhancer-regulated by PAX8, rather than promoter-regulated [37]. An example of a PAX8 target gene (and also HIF) is ferroxidase ceruloplasmin (CP), implicated in the iron-metabolic pathway in RCC tumourigenesis [37]. CP is also a marker of refractory disease and low survival in RCC patients in addition to being a predictor of PAX8 activity [37]. Table 1 summarizes examples of CRC TFs discussed in this section.

### 2.5. Liposarcoma

Liposarcomas (LPSs), or soft tissue sarcomas, are mesenchymal tumours that account for 20% of adult sarcomas [66]. Somatic abnormalities in LPS tumours comprise overexpression of CDK4 and MDM2, and 12q13–15 amplification [67]. Four LPS subtypes have been identified; well-differentiated (WDLPS), myxoid (MLPS), pleomorphic (PLPS) and de-differentiated (DDLPS), the latter three comprising most high-grade cases; PLPS and DDLPS mainly lead to disease relapse post-treatment, while MLPS displays better prognosis [68].

Charting H3K27ac modifications of LPS (DDLPS and MLPS) cell lines and primary tissue, mesenchymal stem cells and mature adipocytes, revealed that some SEs are retained from the adipogenesis programme (e.g. *FOSL2*). By contrast, SEs of definitive adipocyte genes are ablated (e.g. *CEBPA* and *PPARG*) while there is *de novo* establishment of SEs related to genes associated with transformation (e.g. *MYC, CDK6* and *JUN*) [38]. In these LPS samples, the SEs preferentially used are those associated with tumourigenesis, including cell migration, angiogenesis and other developmental processes [38]. Finally, a low-to-moderate overlap was observed between DDLPS and MLPS SEs in primary tissue and cell lines [38].

The defining factor in the MLPS CRC is a fusion oncogene resulting from the t(12;16)(q13;p11) translocation, forming a hallmark MLPS FUS-DDIT3 fusion which functions as a TF [69,70]. FUS-DDIT3 is disproportionately distributed in the genome, especially in SE regions contributing to deregulated gene expression and an aberrant epigenetic landscape. One interesting observation in this subtype was transcriptional addiction owing to preferential SE association with genes regulating RNA-Pol2 activity. Consistent with this, close to 9% of FUS-DDIT3 bound to promoters with high RNA-Pol2 activity [38]. When present, a double H3K27ac and FUS-DDIT3 mark led to high basal expression levels (e.g. *FST* and *IL8*), displaying its potential for corruption of epigenetic landscapes. A known group of interactors with histone acetylation marks of SE regions are bromodomain and extra terminal domain proteins (BET) [71]. Consistent with the notion that oncogenic fusion TFs hijack BET proteins to activate malignant transformation, substantial co-localization and co-operation between FUS-DDIT3 and the BET protein BRD4 has been detected in MLPS [11,38]

CRCs associated with DDLPS comprise FOSL2, MYC and RUNX1, whose maintenance is dependent on BET proteins. Marked co-occupancy of RUNX1 and FOSL2 activates a network of targets involved in the pathogenesis of liposarcoma and malignant growth [38]. Specifically, FOSL2 and RUNX1 proteins co-occupy the SE regions of all described CRC TFs in this LPS subtype. These genes collectively maintain the expression of SNAI2, indicated in EMT and proliferative capacity, and a potential prognostic marker for this subtype. Higher SNAI2 is also linked to shorter disease-free survival (DFS) in DDLPS patients [38]. Finally, demonstrating the dependency of the DDPLS CRC on BRD4, depletion of BRD4 attenuated distant metastasis [38]. Table 1 summarizes examples of CRC TFs discussed in this section.

### 2.6. Prostate cancer

Prostate cancer is one of the major causes of cancer-related deaths in men [72]. The androgen receptor (AR) dictates the transcriptional output that promotes proliferation and survival of prostate cancer cells. Studies focused on dissecting the mechanisms of AR-centred prostate cancer development reveal that AR not only regulates gene expression but also regulates higher-order chromatin configuration [73]. More specifically, a study [39] identified that 55% of AR binding sites function as anchors that mediate duplex and complex AR-associated chromatin interactions (AR_anchor_), while the remaining 45% did not participate in chromatin interaction (AR_alone_). There was a two-fold enrichment of androgen upregulated genes in AR_anchor_ regions compared with AR_alone_ regions, which highlights that long-range chromatin looping may be pivotal to AR regulatory functions [39].

TFs can interact with nuclear hormone receptors such as the AR to govern different aspects of transcription and chromatin regulation [74]. A recurrent fusion gene in prostate cancers, ERG (erythroblast transformation-specific related gene), was shown to interact and collaborate with AR through chromatin looping [73,74]. The ERG interactome, including ERG-associated long-range chromatin, is a collaborative component of higher-order AR-associated chromatin structure and is involved in co-regulating subtypes of AR target genes in prostate cancer. For instance, this study detected intertwined ERG-associated and AR-associated chromatin loops in relation to genes or gene clusters such as *FKBP5, VCL, KLK* family*, EAF2* and *SLC15A2-ILDR1* [39].

AR and ERG co-bind to regulatory sites associated with long-range chromatin interactions (AR^+^ERG^+^_anchor_). These sites have been shown to be associated with enhancer activity, TF binding motifs and bi-directional transcription [39]. Further, these AR and ERG-associated highly connected hubs co-localized with sites for binding of epigenetic regulators/histone remodelling factors and lncRNAs [39]. With regard to co-localization of epigenetic regulators/histone remodelling factors with distinct AR-ERG transcriptional network, three distinct genomic signatures were identified: (i) FOXA1, EZH2 and HDAC3 that are enriched with AR^+^ERG^+^_anchor_ sites; (ii) HDAC1, BRD2, BRD3 and BRD4 that are enriched with AR^-^ERG^+^_anchor_ and AR^-^ERG^+^_alone_ (ERG in the absence of AR); and (iii) POLR2A, HDAC2 and GAPBPA that are enriched with AR looping but not AR^+^ERG^+^_alone_ and AR^+^ERG^-^_alone_ [39].

With respect to IncRNAs, one potential function of AR and ERG chromatin looping may be to allow interactions between lncRNA and its target gene. For instance, manipulating three lncRNAs identified in association with the *PMEPA1* locus (*PCAT43, PCAT61* and *PCAT76*) led to a reduction in androgen-triggered expression of the gene [39]. One other example of the clinical relevance of AR and ERG chromatin loops is the link detected between a prostate cancer GWAS SNP, rs9364554, located in the intron of *SLC22A3* within an AR and ERG loop anchor. This loop also connects this SNP with *SLC22A2* in the vicinity [39]*.* Table 1 summarizes examples of CRC TFs discussed in this section.

### 2.7. Gastrointestinal stromal tumour

Gastrointestinal stromal tumour (GIST) is a common soft tissue sarcoma, originating from interstitial cells of Cajal (ICC) [75]. The ICC lineage is reliant on KIT and ETV1 for specification and survival, whereby KIT and ETV1 function as signalling and lineage-specific regulators, respectively [75,76]. During development, the transcriptional input required for ICC lineage specification constitutes KIT activation by KIT ligand and consequent MAPK-mediated stabilization of ETV1 protein, establishing lineage specification [75]. In the pathological context, mutant KIT stabilizes ETV1 (through aberrant MAPK signalling activation), while in turn, ETV1 promotes mutant KIT expression, forming a divergent positive feedback loop fuelling the process of tumourigenesis [40].

FOXF1, a member of the fork-head family of transcription factors, is specifically expressed in GIST and directly regulates the transcription of *KIT* and *ETV1*. In turn, FOXF1 and ETV1 both regulate KIT, although FOXF1 regulation of KIT is significantly stronger owing to the regulation of both chromatin accessibility and the ETV1 cistrome [40]. This evidence may support the pre-existence of this regulatory pattern between KIT and FOXF1 in non-oncogenic ICC development, highlighting similarities between physiological and pathological development.

FOXF1 also co-localizes with ETV1 to regulate ICC/GIST lineage-specific gene expression by maintaining open chromatin structure and enhancers, as well as the recruitment of ETV1 to lineage-specific enhancers. Examples of ETV1-dependent ICC/GIST lineage-specific gene networks regulated by FOXF1 include *DUSP6, GPR20* and *ANO1* [40].

With respect to FOXF1 regulation, KIT or MAPK pathway perturbations do not significantly affect the expression of *FOXF1*, placing it at the top of a regulatory hierarchy for GIST. Finally, FOXF1 is required for GIST cell cycle progression, tumour growth and maintenance [40]. Table 1 summarizes examples of CRC TFs discussed in this section.

### 2.8. Medulloblastoma

Medulloblastoma, a malignant paediatric brain tumour arising from the cerebellum, medulla and brain stem, is categorized into four clinically and biologically distinct subgroups [77]. These four core subgroups, WNT, SHH, group 3 and group 4, are classified based on their inherent differential and discriminatory transcriptional profiles. The WNT and SHH subgroups are named based on the activity of the respective pathways, and groups 3 and 4 display regulatory similarities [78] but present diverse phenotypes and express GABAergic and glutaminergic cell-type characteristics, respectively [21,77]. In addition to somatic alterations in driver genes such as *MYC* (group 3), *KDM6A* (group 4) and *GFI1/ GFI1B* (group 3 and 4) [21,77,79], epigenetic modulation may influence transcriptional programming specific to subgroups [80].

The computational reconstruction of SE and enhancer mapping for 28 medulloblastoma primary tissue has been used to dissect differential group 3 and 4 CRCs [21]. This mapping approach identified large SEs associated with cerebellum-specific TFs, *ZIC1* and *ZIC4*, and SEs associated with medulloblastoma driver genes and epigenetic modulators, such as *GLI2*, *MYC* and *OTX2* [21]. On a subgroup level, SEs were then inferred to regulate *ALK* in the WNT group, *SMO* and *NTRK3* in the SHH group, *LMO1*, *LMO2* and *MYC* in group 3, and *ETV4* and *PAX5* in group 4 [21]. This group-specific SE allocation was based on an unbiased hierarchical clustering strategy of SEs across the samples analysed. One key observation in the study was that SE patterns observed differed substantially between medulloblastoma primary tissue or cell lines highlighting regulatory and CRC component dissimilarities [21]. This study also identified core TFs implicated in establishing medulloblastoma group identity including HLX (group 3), LMX1A (group 4) and LHX2 (shared between groups 3 and 4), providing some evidence towards the cell-of-origin of these disease groups [21]. In terms of functional pathway enrichment, TGFβ signalling and neuronal transcriptional regulators were enriched in groups 3 and 4, respectively [21]. Table 1 summarizes examples of CRC TFs discussed in this section.

### 2.9. Chronic lymphocytic leukaemia

Chronic lymphocytic leukaemia (CLL) is a highly heterogeneous B-cell haematological malignancy with low cure rates. A spectrum of genomic alterations in this malignancy have been identified, including segmental chromosomal alterations, copy number alterations and somatic nucleotide alterations, while 13q deletion is the most recurrent alteration [81,82]. The CLL-specific CRC is centred on PAX5, a TF that promotes lymphomagenesis by activating signalling pathways indicated in B-cell signalling, and the knockdown of this gene results in dramatic effects on B-cell proliferation and development [41,83].

In a study aimed at dissecting CRCs in primary CLL and normal B cells (NBCs), SEs with exceptionally high H3K27ac marks (42% of all H3K27ac marks globally) were discovered in proximity to genes involved in CLL pathobiology, including *CXCR4, CD74, PAX5, CD5, KRAS* and *BCL2* [41]. This high proportion of H3K27ac at these few loci of total global H3K27ac activity concomitant with open chromatin structure (tested by ATAC-seq) demonstrates the dominance of these SEs in regulating transcriptional output. For instance, the SE of the *BCL2* gene that is usually upregulated in CLL, open chromatin structure and broad H3K27ac signals were detected [41]. The SE of *CTLA4*, encoding a T-cell inhibitory checkpoint effector, also displayed strong H3K27ac signals. The NBC samples used in this study showed 230 SEs, including SEs proximal to *BACH2* and *BANK1*, known to play roles in lymphoma suppression [41,84]. Further, despite samples displaying substantial heterogeneity, a core of large SEs displayed regulatory conservation among a subset of the CLL patient samples in loci pertinent to *KRAS, CD5, PAX5, CXCR4, BCL2* and *CD74* [41]. Finally, this study defines an enhancer-based CRC analysis system. Specifically, for TFs associated with top-ranked enhancers, inward TF enhancer binding by other TFs and outward binding of the TF of interest to their extended enhancer network were assessed. This information was processed to describe ‘cliques’ of auto-regulatory TFs [41]. At least four representative cliques were defined: CLL-2, CLL-3, CLL-8 and CLL-11. For instance, TFs constituting the CLL-2 clique include PAX5, ETV6, TCF3, IRF2, MEF2D, ELF1, KLF13, JUND, FOXP1, IRF1 and IRF8 [41]. Highly connected CLL and NBC TFs across samples comprised PAX5 and the IRF family in addition to FOXP1, RARA and ETS1 [41]. Table 1 summarizes examples of CRC TFs discussed in this section.

### 2.10. T-cell acute lymphoblastic leukaemia

For T-cell acute lymphoblastic leukaemia (T-ALL), malignant transformation gives rise to leukaemic cells owing to deregulated thymic differentiation programmes [85]. The oncogenic TF, TAL1, is crucially involved in the pathogenesis of T-ALL cases and has been shown to collaborate with other TFs to form a CRC. This CRC comprises TAL1, HEB, E2A, LMO1/2, GATA3 and RUNX1 in T-ALL representative cell lines, such as Jurkat and CCRF-CEM [42]. A high coincidence of genomic site occupation was observed in this study between TAL1 and other CRC TFs including LMO1/2, GATA3 and RUNX1. In these two cell lines, three different classes of regulatory elements were identified: group 1 (concordant enrichment for TAL1 complexes), group 2 (mainly GATA3 occupation) and group 3 (mainly RUNX1 occupation). In terms of the presence of identified binding motifs, these were, for group 1, E-box, GATA, RUNX and ETS, for group 2 GATA and ETS motifs, and for group 3 RUNX, ETS and SP1 [42].

In summary, TAL1 forms an auto-regulatory loop with GATA3 and RUNX1, and they occupy regulatory regions of their own and each other's genes. TAL1 initiates this auto-regulatory loop, and the sustained upregulation of GATA3 and RUNX1 by TAL1 may contribute to reinforcement of the malignant programme in T-ALL [42]. Further, TAL1 positively regulates the expression of a network of target genes in collaboration with GATA3 and RUNX1 [42].

Target genes of TAL1 include *TRIB2* and *MYB* whereby the former regulates cell survival in TAL1-positive T-ALL cells, while the latter is a transcriptional regulator driving normal and malignant blood haematopoiesis [86]. *MYB* is induced by TAL1 and in turn, MYB co-regulates a subset of TAL1 target genes, stabilizes and reinforces the TAL1 oncogenic programme [42]. One example for collaboration between TAL1 and MYB in TAL1-positive T-ALL cells is that the enhancer region of TAL1 can be targeted by numerous somatic alterations which then form new MYB binding sites and SEs, effectively extending the outreach of MYB [18]. An example of negative and positive regulation in T-ALL is the TAL1, HEB and H2A regulatory network. TAL1, HEB and H2A coordinately regulate target genes. Of these target genes, a subset is directly activated by TAL1 but repressed by HEB and H2A [42]. Table 1 summarizes examples of CRC TFs discussed in this section.

## 3. CRCs and drug development

**Figure 2.**
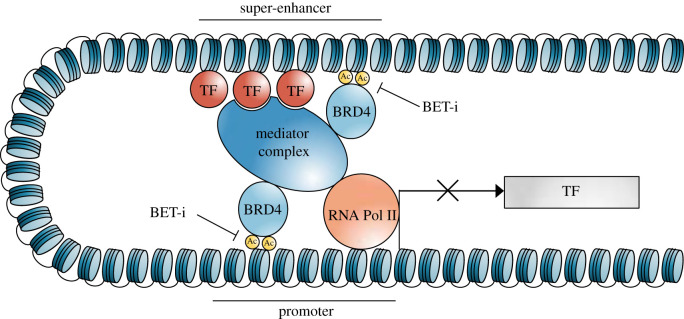
BET inhibitor treatment represses transcription of super enhancer-associated transcription factors. BET proteins (including BRD4) regulate chromatin and RNA polymerase accessibility to the gene of interest. BET inhibitors such as JQ1 can induce disruption of SEs and specific transcription elongation defects and inhibition by displacing BRD4.

The dissection of regulatory networks associated with cell identity in cancer facilitates a better understanding of the malignancy and the identification of appropriate treatment strategies. CRCs provide a framework for the identification and potential targeting of oncogenic CRC TFs, transcriptional co-activators, SEs and SE-associated co-activators and modulators as justifiable avenues of targeting. One example of targeting master regulator TFs for therapeutic gain is in GIST. This cancer is highly resistant to standard chemotherapy, and is instead sensitive to specific targeting of KIT and ETV1 lineage-specific CRC TFs [87,88]. Further, CRC TFs recruit acetylation writers such as CBP/p300, readers such as BRD4 and erasers such as HDACs and other factors to construct SEs [8,22]. BRD4 and related proteins have been shown to occupy large numbers of enhancers, especially SEs [11,15]. Due to this association, SEs may be sensitive to drugs that target BET domain regulators and kinases involved in transcription [15,89]. Despite the broad presence of BET proteins across thousands of enhancers, inhibition of these proteins (for instance the inhibition of BRD4 by the BET-bromodomain inhibitor JQ1), has led to specific targeting in multiple cancers, revealing cancer dependencies. In multiple myeloma, JQ1 treatment led to specific MYC inhibition [15] (figure 2), while in CLL, BET inhibition led to the downregulation of multiple survival pathways involved in CLL biology [90]. This pattern was also observed in diffuse large B-cell lymphoma (DLBCL), in which SEs of oncogenic and lineage-specific CRCs showed particular sensitivity to BET inhibition [11].

In addition to gene or gene network targeting, BET protein inhibition may be explored to sensitize cases of relapse and treatment resistance. For instance, in solid tumours such as LPS, targeting BET proteins using ARV-825, a BET protein degrader, can provide advantages in overcoming trabectedin resistance [38]. In terms of cellular effects, BET protein inhibition and depletion mainly triggers apoptosis or cytotoxic effects in cancers, including osteosarcomas and breast cancer [91,92].

One other outcome of chemical targeting of SEs is to understand SE driven transcriptional addiction in cancers. In multiple myeloma, JQ1 treatment more dramatically affects SEs and SE-associated genes compared with typical enhancer-associated genes [15]. Cancer addiction to CR transcription has been described in RMS, in which the PAX3-FOXO1 fusion protein activates SEs to activate the expression of other CR TFs in a feed-forward manner, leading to high levels of CR TF expression [22]. Consistent with transcriptional addiction, the selective disruption of CR transcription was achieved by targeting the acetylation axis in this cancer [22]. Specifically, this study showed that co-inhibition of HDAC1, HDAC2 and HDAC3 halts CR transcription by interfering with chromatin accessibility and looping [22]. In conclusion, understanding the dependency and mechanistic connections between BET proteins and deregulated programmes and enhancer states can provide avenues for target identification and therapeutic gain.

## 4. Similarities between CRC models in the wider context of human cancer

**Figure 3.**
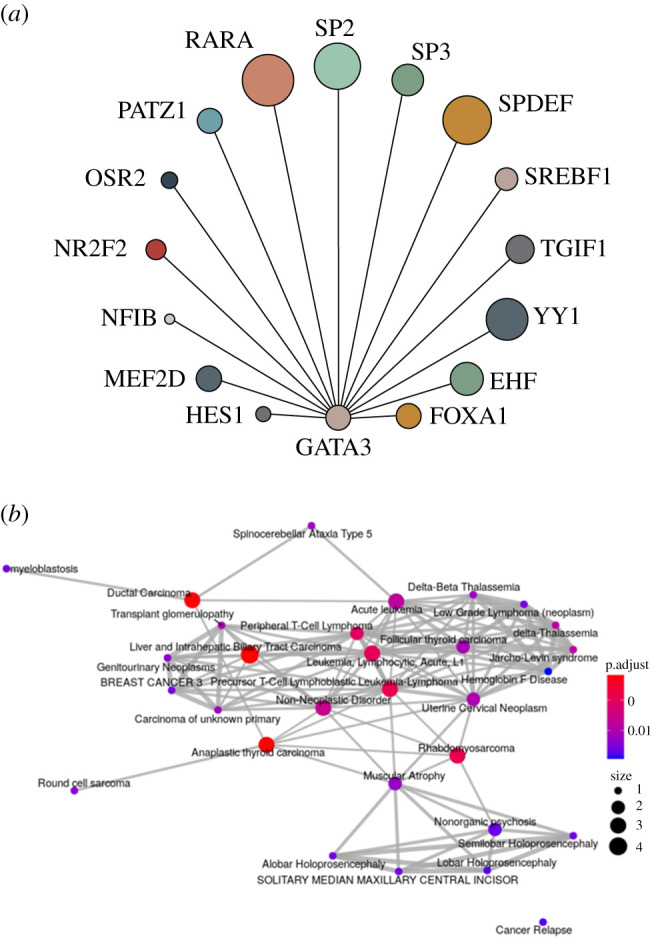
(*a*) GATA3 is indicated in the CRC model of the ZR-75-1 breast cancer cell line. CRC model output from dbCoRC for GATA3 in the ZR-75-1 breast cancer cell line involving GATA3 regulation of 15 target TFs (by binding to their SEs). The circumference of each target TF circle is proportionate to the number of GATA3 binding sites identified in SEs of the gene encoding this target TF. (*b*) Using DisGeNet without multiple testing corrections, the CRC model TFs in (*a*) show an association with several cancers and other diseases. Each link represents the number of overlapping genes annotated to each term, and size represents the number of genes annotated to each term.

The CRC TFs identified in this study, although displaying specific functions in each cancer's CRC, may indeed be involved in gene regulatory networks in a spectrum of other human cancer cell lines and primary tissues. The dbCoRC database permits the collation of information concerning cell or tissue expression of a given CRC TF, upstream and downstream targets of this TF within the CRC model, SE genomic coordinates and the number of TF binding sites within the SE of the targets (CRC TFs) [19]. Here, we have used this tool to further study the CRC TFs indicated in the 10 cancer types discussed in this review in other cell lines. Table 2 outlines reviewed CRC TFs indicated in other human cell lines and primary tissue, and the CRC model formed. For instance, FOXP1 and ERG reviewed in the context of CLL and prostate cancers, respectively, are both indicated in the CRC model of a colorectal cancer cell line, COLO320 (ASCL2, DBP, ERG, FOXG1, FOXP1, MEIS1, OSR1, SOX5, SP1, TBX2, TEAD1, TFAP2C and TFAP4). Another example is GATA3, which has been reviewed in this article in NB and T-ALL, and has also been identified in the regulatory networks of a breast cancer cell line (ZR-75-1) [19,31,42]. This regulatory network comprises TFs such as: EHF, FOXA1, GATA3, HES1, MEF2D, NFIB, NR2F2, OSR2, PATZ1, RARA, SP2, SP3, SPDEF, SREBF1, YY1 and TGIF1 (figure 3*a*). The CRC model proposed by dbCoRC for GATA3 in breast cancer was further processed using DisGeNet to test the association of these TFs with other cancers and other diseases (figure 3*b*) [93,94]. Using this programme and without correction for multiple testing, strong associations with several cancers were identified. Each link represents the number of *overlapping* genes annotated to each term, and size represents the number of genes annotated to each term. These data highlight the importance of understanding and comparing TFs across a wider spectrum of cancer cell lines and primary tissue, with the objective of the discovery of overlapping and non-overlapping functions and mechanisms.

**Table 2.** Summary of the CRC network data extracted from dbCoRC database for CRC TFs discussed in this review. CRC TFs discussed in this study were investigated using dbCoRC database to identify the differential utility of these TFs in CRCs models of other human cancer cell lines and primary tissue. The example provided for the implication of the TF in other cancer cell line or primary tissue represents one of many examples provided by this database.

CRC TF/ malignancy	other cancer cell lines or primary tissue	examples of upstream/downstream TFs within the CRC model in this cell line or primary tissue
PHOX2B/ NB	NCI-H82 (SCLC)	OTX2, SREBF1, TEAD1, MYC, NHLH1, NR2F6, PHOX2B
GATA3/NB and T-ALL	ZR-75-1 (breast carcinoma)	EHF, FOXA1, GATA3, HES1, MEF2D, NFIB, NR2F2, OSR2, PATZ1, RARA, SP2, SP3, SPDEF, SREBF1, YY1, TGIF1
SOX2/GBM	NCI-H69 (SCLC)	BARHL1, DLX1, ETS1, FOXA1, SOX2, FOXG1, INSM1, KLF13, KLF7, MSX2, NR2F1, SP8, TCF4, TEAD1
MYOD1/RMS	RH18 (RMS)	ARID3A, FOXL1, GLI1, GLI3, HOXC9, IRF1, MAFK, MYOD1, RARA, RXRA, SMAD3, TBX1, TEAD3, VDR
MYOG/RMS	RD (RMS)	ETV4, GLI3, HOXC10, HOXC9, HOXD8, KLF7, MYOD1, MYOG, RUNX1, SMAD3, SOX8, TCF7L2, ZNF219
MYC/LPS	NCI-H82 (SCLC)	MYC, NHLH1, NR2F6, OTX2, PHOX2B, SREBF1, TEAD1
RUNX1/LPS and T-ALL	T20020720 (gastric cancer)	EHF, ELF3, ETS2, IRF1, IRF2, KLF13, KLF5, MAFF, MEIS1, NR4A1, PRDM1, RREB1, RXRA, SMAD3, SOX13, TCF7L2, RUNX1
ERG/prostate	COLO320 (colorectal cancer)	ASCL2, DBP, ERG, FOXG1, FOXP1, MEIS1, OSR1, SOX5, SP1, TBX2, TEAD1, TFAP4, TFAP2C
PAX5/CLL	SU-DHL-6 (diffuse large B-cell lymphoma)	ARID5B, CUX2, ELF1, MAX, PAX5, SMAD3
ETV6/CLL	T2000085 (gastric cancer)	BCL6, BHLHE40, ETS1, ETV6, GLIS3, HIVEP2, IKZF1, IRF2, KLF7, MEF2D, MEIS1, NR2F2, RARA, RREB1, RUNX1, SMAD3, ZBTB16
IRF2/CLL	HCC1954 (breast cancer)	ELF3, FOXI1, HES1, IKZF2, IRF2, NFIA, PBX1, PITX1, SP3, STAT4, TFAP2A, TP63
ELF1/CLL	COLO205 (colorectal cancer)	ASCL2, BARX2, BHLHE40, DLX2, EHF, ELF1, ELF3, FOS, FOXB1, HES1, HNF1B, IRF1, IRF8, KLF5, MYB, PDX1, PITX1, RREB1, RUNX1, RUNX3, SMAD3, SREBF1, TCF7, TCF7L2, TEAD1
KLF13/CLL	T2001206 (gastric cancer)	BHLHE40, ELF3, ETS1, ETS2, ETV6, HIF1A, IRF1, IRF2, KLF5, KLF13, MEIS1, PRDM1, RREB1, RUNX1, SMAD3, TCF7L2, TGIF1
FOXP1/CLL	COLO320 (colorectal cancer)	ASCL2, DBP, ERG, FOXG1, FOXP1, MEIS1, OSR1, SOX5, SP1, TBX2, TEAD1, TFAP2C, TFAP4
NFATC1/CLL	HBL1 (diffuse large B-cell lymphoma)	BACH2, EBF1, ETS1, ETV6, FOXP1, HES1, IKZF1, IRF2, IRF4, IRF8, MAX, MEF2A, MEF2D, NFATC1, NR3C1, PAX5, POU2F2, RORA, RUNX1, TCF4, TBX15, TFEB
KLF12/CLL	COLO741 (colorectal cancer)	EGR3, EN2, ETS1, KLF12, NR4A1, NR4A2, PKNOX2, RARA, RREB1, SMAD3, SNAI2, SP1, SREBF1, TBX2, TEAD1, TGIF1
JUN/CLL	MiaPaca2 (pancreatic adenocarcinoma)	EHF, HOXB6, JUN, MYBL1, MYC, NR2F2, NR5A2, RXRA, SHOX2, SMAD3, SREBF1, TBX4, TP63, WT1

## 5. Methodologies that facilitate the understanding of CRCs

Next-generation technologies have allowed shifting from inter-patient tumour variability to the precise characterization of intra-tumour genetic, genomic and transcriptional heterogeneity via multi-regional bulk tissue NGS. Emerging single-cell transcriptomics, coupled with NGS, allow novel strategies for therapeutic response prediction and drug development. The regulatory mechanisms that govern the transcriptome and the expression of these regulatory circuits are now being investigated using WGS to identify non-coding mutations and chromatin profile using ChIP-seq (chromatin immunoprecipitation followed by sequencing), 4-C (circulized chromatin conformation capture), ChIA-PET (chromatin interaction analysis with paired-end tags) and ATAC-seq (assay for transposase-accessible chromatin followed by sequencing) [95,96]. Understanding regulatory networks at single-cell resolution has empowered efforts to decipher cancer heterogeneity, differential resistance to therapy patterns and hierarchical classification, for instance, in breast cancer [97]. Here, we briefly elaborate on each method.

ChIP-seq is a technique allows the detection of TF binding profiles and histone modifications, including the H3K27ac marks that signify SEs. The challenge with this technique is obtaining a highly specific antibody [8].

4C-seq is an update of the chromosome conformation capture (3C) coupled to sequencing (Hi-C) method that quantifies contact frequencies of DNA based on nuclear proximity, and reveals chromatin folding and configuration patterns [98]. 4C-seq takes into account domains of contact and inter-domain contact of a specific genomic site within genome sequences [99]. The main limitation of 4C is technical biases due to coverage of *cis* and *trans* chromosome interactions and the use of restriction enzymes [100,101]. ChIA-PET detects chromatin interactions associated with a protein of interest. This method is unbiased and relies on the premise that proximal DNA sequences from the same cross-linked molecular complex may be ligated, offering enhanced resolution and throughput compared with previous techniques [100]. The limitations of ChIA-PET include the requirement for substantial starting material due to the sequence of experimental steps. An improved adaptation of this method is proximity ligation-assisted ChIP-seq (PLAC-seq), which features shifting forward of the ligation step. Briefly, in this method, *in situ* proximity ligation is performed prior to lysis of the nuclei, significantly reducing the required input material and improving the efficacy and accuracy over ChIA-PET [102]. Another improved method of detecting chromatin conformation mediated by a protein of interest that addresses limitations of ChIA-PET is HiChIP. This method also relies on *in situ* establishment of DNA contacts prior to lysis of nuclei. Subsequently, ChIP and on-bead library generation is carried out followed by paired-end sequencing, revealing the long-range interactome of the protein of interest [103]. A significant drawback of HiChIP is the effect of sequencing depth on the accuracy of detected interactions. Gryder and colleagues address this drawback by introducing AqUa-HiChIP [104]. This method circumvents the limitation of HiChIP by absolute quantification of chromatin interactions. Briefly, this method relies on a previously defined ratio of formalin-fixed nuclei of two different origins (for instance mouse versus human nuclei). The nuclei are lysed, and upon incorporation and ligation of biotin-dATP, shearing is performed. Subsequently, ChIP, biotin capture and paired-end sequencing are performed [104]. Human chromatin interactions are then normalized to those of the mouse genome on the grounds of paired-end tag counts, allowing more accurate quantification of these interactions. Alongside the experimental method, this group also provides a streamlined bioinformatics analysis platform coupled to this method [104].

ATAC-seq assays the transposase accessibility of chromatin coupled with next-generation sequencing. It relies on the insertion of sequencing linkers by a hyperactive Tn5 transposase enzyme. Sequencing of the linker attached to reads reveals regions of chromatin accessibility and offers higher sensitivity compared with other techniques such as DNAse-seq. Limitations in streamlined bioinformatics analysis pipelines may be a challenge with this technique [105]. Finally, single-cell-resolution ATAC-seq can inform areas of chromatin accessibility and shed light on developmental processes [106].

## 6. Conclusion

This review summarizes CRC TF members associated with SEs in a range of liquid and solid cancers. CRC TFs create and maintain cell-type specific regulatory programmes and define cell identity, a process that is deregulated in many cancer subtypes. Specific TFs play important roles in forming CRC networks in several types of cancer cell lines and primary tissues, suggesting similar yet divergent mechanisms and players involved in regulatory processes. Reconstruction of CRCs in cancer cell lines and tissue, obtained by leveraging genomic technologies, will facilitate the understanding of deregulation of biological processes in carcinogenesis and support the reconstruction of a blueprint pertaining to the identity of a cancer. Consistent with this, transcriptional addiction is emerging as an important novel drug vulnerability in cancers. Therefore, understanding components of CRCs, associated proteins and regulators can provide opportunities for targeting of these components for therapeutic advantage.

## Data accessibility

This article has no additional data.
